# The oldest peracarid crustacean reveals a Late Devonian freshwater colonization by isopod relatives

**DOI:** 10.1098/rsbl.2021.0226

**Published:** 2021-06-16

**Authors:** N. Robin, P. Gueriau, J. Luque, D. Jarvis, A. C. Daley, R. Vonk

**Affiliations:** ^1^ School of Biological, Earth and Environmental Sciences, University College Cork, Distillery Fields, North Mall, Cork T23N73K, Ireland; ^2^ Institute of Earth Sciences, University of Lausanne, Géopolis, 1015 Lausanne, Switzerland; ^3^ Museum of Comparative Zoology and Department of Organismic and Evolutionary Biology, Harvard University, 26 Oxford Street, Cambridge, MA 02138, USA; ^4^ Department of Taxonomy and Systematics, Naturalis Biodiversity Center, 9517, 2300 RA Leiden, The Netherlands; ^5^ Institute for Biodiversity and Ecosystem Dynamics, University of Amsterdam, 94240, 1090 GE Amsterdam, The Netherlands

**Keywords:** Peracarida, Palaeozoic, Eumalacostraca, Famennian, Kiltorcan, Ireland

## Abstract

Peracarida (e.g. woodlice and side-swimmers) are, together with their sister-group Eucarida (e.g. krill and decapods), the most speciose group of modern crustaceans, suggested to have appeared as early as the Ordovician. While eucarids' incursion onto land consists of mainly freshwater and littoral grounds, some peracarids have evolved fully terrestrial ground-crawling ecologies, inhabiting even our gardens in temperate regions (e.g. pillbugs and sowbugs). Their fossil record extends back to the Carboniferous and consists mainly of marine occurrences. Here, we provide a complete re-analysis of a fossil arthropod—*Oxyuropoda—*reported in 1908 from the Late Devonian floodplains of Ireland, and left with unresolved systematic affinities despite a century of attempts at identification. Known from a single specimen preserved in two dimensions, we analysed its anatomy using digital microscopy and multispectral macroimaging to enhance the contrast of morphological structures. The new anatomical characters and completeness of *Oxyuropoda*, together with a phylogenetic analysis with representatives of all major Eumalacostraca groups, indicate that *Oxyuropoda* is a crown peracarid, part of a clade including amphipods and isopods. As such, *Oxyuropoda* is the oldest known species Peracarida, and provides evidence that derived peracarids had an incursion into freshwater and terrestrial environments as early as the Famennian, more than 360 Ma.

## 1. Introduction

Peracarid crustaceans (e.g. woodlice, opossum-shrimps, side-swimmers and comma-shrimps) are eumalacostracans that have diverged parallel to eucarids (shrimps, lobsters and crabs) to produce the most modern crustacean diversity (67 000 described species) [1–3]. In peracarids, the most speciose groups are amphipods (side-swimmers; approx. 10 000 species) and isopods (approx. 10 000 species), forming 20% of the diversity inhabiting rivers and lacustrine environments. A third of the isopod species are widespread terrestrial crawlers known as woodlice—common inhabitants of temperate gardens (e.g. pillbugs and sowbugs) [1–3]. While studies of more inclusive peracarid clades [4–9] did not suggest a diversification age for the group, that of the comparatively late-diverging peracarid order Isopoda is estimated by molecular analyses to have occurred during the Middle to Late Ordovician (approx. 455 Ma) [10,11], implying a long Palaeozoic history of Peracarida. However, reconciling the estimated Ordovician molecular time divergence of peracarids with their fossil record has been challenging [12,13], since the earliest fossil peracarids are of Early to Late Carboniferous age, consisting of a tanaid [14,15], some putative Mid-Carboniferous stem comma-shrimps [16,17], and a Late Carboniferous isopod [18,19].

A crustacean-looking arthropod, found in the early twentieth century from the Famennian (Late Devonian) of Ireland [20], has long been suspected of being an isopod-related animal. Reported from an undoubtedly freshwater assemblage, its affinities, if they could be determined, would help clarify the terrestrial evolution of Pancrustacea [20,21]. Its general aspect, reminiscent of the extant *Ligia*, a coastal terrestrial oniscoid woodlouse, earned it its name *Oxyuropoda ligioides* Carpenter and Swain, 1908. In addition to its round and short head fused to larger thoracic segments, it possesses a tapering and short pleon, with curved and pointed lateral pleurae on the abdominal segments, typical of isopods. However, its anatomy reveals six thoracic segments instead of the usual seven in isopods. Six such segments exist in other peracarids but these are very different in shape [22]. From its discovery to its last observation in 1985 [23] the fossil has successively been assigned to peracarids [20,24–29], phyllocarids [29], euthycarcinoids [30], arachnomorphs [31–33] or other arthropods [30–35]. Here, we re-analyse the visible and hidden anatomy of this continental (freshwater) arthropod using a combination of standard light photography and newly developed luminescence-based imaging methods, and undertake a cladistic analysis to investigate its position within malacostracan crustaceans.

## 2. Material and methods

### (a) *Oxyuropoda* material and Kiltorcan Hill age

*Oxyuropoda ligioides* consists of a single specimen (part and counterpart; holotype NMING : F7633) housed in the palaeontological collection of the National Museum of Ireland, Dublin—Natural History, and recovered from Kiltorcan Hill, County Kilkenny, Ireland. Besides *O. ligioides*, Kiltorcan Hill outcrops have yielded a range of continental freshwater-type organisms, including lycophytes, progymnosperms, green algae *Bythotrephis*, freshwater bivalves, placoderms [20,36] and fragments of eurypterids [37–39]. Carpenter & Swain [20] reported *Oxyuropoda* from what can be recognized as the *Classic* or *Old Quarry* of Kiltorcan [21,36,40]. The corresponding strata are encompassed by outcrops stratigraphically extending both below and above them, that do not yield macrofossils of the same quality and diversity, but miospore assemblages aiding in the age dating of the entire sequence. The most extensive outcrop to be found today on the hill is an active quarry referred as the *New Quarry* [36], which yields LE miospore assemblages of the ‘Strunian’ latest Famennian (uppermost Devonian). The topmost outcrop on the hill (*Roadstone Quarry* [21,36,37]) yields a VI miospore assemblage of the lower-most Carboniferous. The strata on Kiltorcan Hill, therefore, straddle the Devonian/Carboniferous Boundary [36], suggesting that the original Kiltorcan locality, located between New and Roadstone quarries horizons, is likely latest Famennian. To retrieve as many anatomical details as possible from this unique specimen, digital microscopy and multispectral macroimaging were carried out at the Institute of Earth Science, University of Lausanne, to enhance morphological contrast and reveal new anatomical features.

### (b) Two-dimensional and three-dimensional digital microscopy

**Figure 1.**
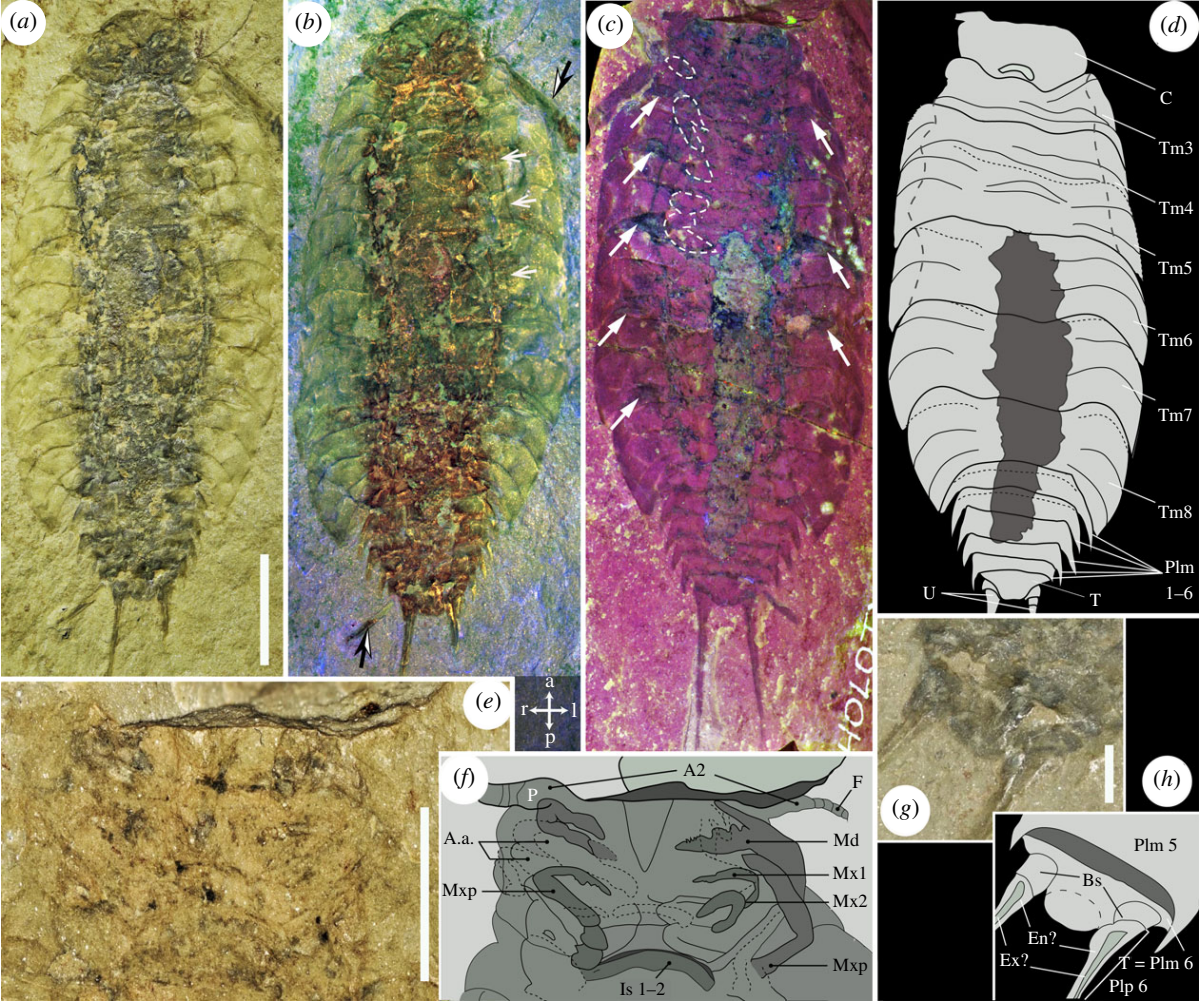
The anatomy of *Oxyuropoda ligioides* Carpenter and Swain, 1908, holotype NMING : F7633. Part (*a*,*b*,*g*,*h*), counterpart (*c*–*f*). (*a*–*d*) Total body. (*e*,*f*) Cephalon close-up. (*g*,*h*) Pleotelson close-up. Artificial lightning using crossed polarizing filters (*a*), digital scanning surface microscopy (*e*,*g*), multispectral macroimaging setting 1 (*b*), setting 2 (*c*), black-and-white arrows = plant remains, white dash-lines = outlines of the visible pereopods, long white arrows = areas of overlapping portions of thoracomeres, short white arrows = identified taphonomic cracks. Interpretative drawings (*f*,*h*), black dash-lines = limits from which pleurae are bending. A.a., Probable additional appendage; A2, antenna; Bs, basipodites; C, cephalon; En?, possible endopodite; Ex?, possible exopodite; F, flagellum; Is 1–2, intersternite 1–2 thickening; Md, possible mandible; Mxp, possible maxilliped; Mx1, possible maxillula; Mx2, possible maxilla; Tm 3–8, thoracomeres 3–8; P, antennal peduncle; Plm 1–6, pleomeres 1–6; Plp 6, pleopod 6; T, telson; U, uropods; l/r/a/p, left, right, anterior, posterior referential. Scale bars = 10 mm (*a*–*d*), 5 mm (*e*), 1 mm (*g*).

A Canon EOS 800D digital SLR camera fitted with an EF-S 60 mm *f*/2.8 Macro lens was used to photograph the specimen under artificial lighting from multiple directions. Polarizing filters at the lens and the light source created crossed polarization that reduced reflections and increased contrast. Digital microscopy images were collected using a Keyence VHX-7000 digital microscope equipped with a VH-ZOOT Macro lens (0×–50× magnification), connected to a VXH-7020 high performance 3.19-megapixel CMOS camera. High-resolution three-dimensional images were collected through an automatic stitching process. Three-dimensional images presented herein in natural (figure 1*e–g*, electronic supplementary material; figure SF1*c*, *e–g*) or false warm–cold (electronic supplementary material, figure SF1*b*) colours were produced through an automatic vertical stacking process that creates three-dimensional surface profiles.

### (c) Multispectral macroimaging

Building on the concept of UV photography [41] and visible light fluorescence imaging [42–44], we collected reflection and luminescence images in various spectral ranges using an innovative imaging setup under development (see [45]). The setup consists of a low-noise 2.58-megapixel back illuminated sCMOS camera (PRIME 95B 25 mm, Photometrics) with high sensitivity from 200 to 1000 nm, fitted with a UV–VIS–IR 60 mm 1 : 4 Apo Macro lens (CoastalOptics) in front of which is positioned a filter wheel holding eight interference band-pass filters (Semrock) to collect images in eight spectral ranges from 435 to 935 nm. Illumination is provided by 16 LED lights ranging from 365 up to 700 nm wavelength (CoolLED pE-4000), coupled to a liquid light-guide fibre fitted with a fibre-optic ring light-guide. As such, more than 90 different illumination/detection couples are available, and the resulting greyscale images can be combined into false colour RGB images to enhance morphological contrasts or reveal new details in a wide range of fossils [45,46]. Stacking, image registration of the different couples, and production of false colour RGB composites were performed using ImageJ. The field of view being smaller than the specimen, images of the full body were produced by producing RGB images at three different positions, which were then stitched together using Image Composite Editor (Microsoft). False colour RGB images presented herein were produced using two settings: (i) (figure 1*b*) red—illumination 435 nm/detection 435 ± 20 nm (reflection), green—illum. 660 nm/det. 650 ± 30 nm (refl.), blue—illum. 435 nm/det. 571 ± 36 nm (luminescence); (ii) (figure 1*c*) red—illum. 385 nm/det. 935 ± 85 nm (lum.), green—illum. 435 nm/det. 571 ± 36 nm (lum.), blue—illum. 435 nm/det. 435 ± 20 nm (refl.).

### (d) Phylogenetic analysis

**Figure 2.**
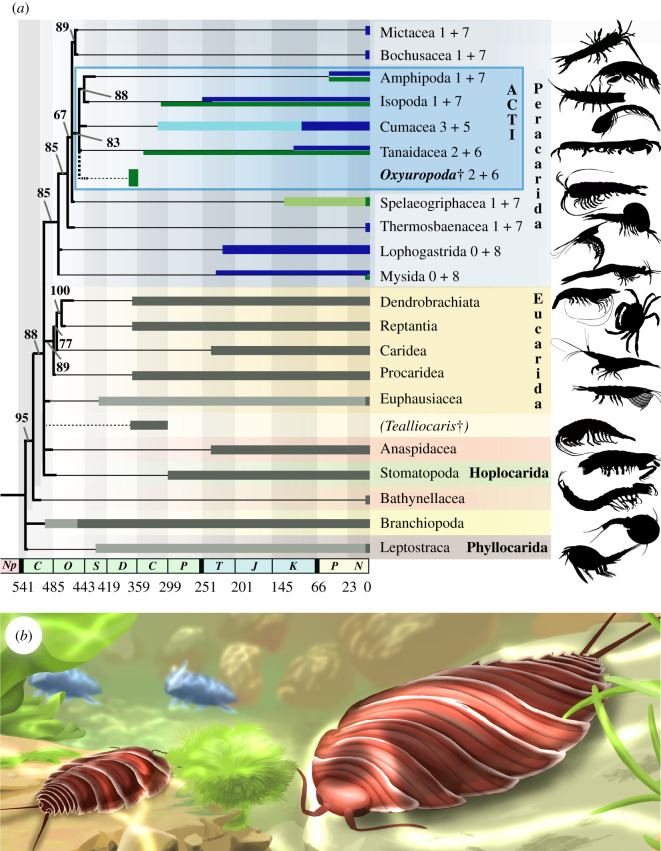
Phylogeny of the Malacostraca including *Oxyuropoda ligioides* Carpenter and Swain, 1908 after [1] and reconstruction of the animal in its freshwater environment in the Late Devonian. (*a*) Bayesian majority-rule consensus topology and branch lengths of the post-burnin sample of trees, plotted on geological times. Branches with posterior probability support less than or equal to 65% collapsed. The obtained tree (black) accommodates here: (i) the Late Ordovician divergence of Isopoda, (ii) the minimal Late Carboniferous divergence of Amphipoda, (iii) the Early Cambrian clade age of Branchiopoda. Light and dark thick lines, respectively, for stem- and crown-groups; grey by default, blue for peracarid marine taxa, green for peracarid freshwater taxa, see [2] for age justification. ACTI = clade grouping Amphipoda, Cumacea, Tanaidacea and Isopoda. 0 + 8: code for number of malacostracan thoracomeres integrated in the cephalon + number of thoracomeres in the thorax (see electronic supplementary material, figure SF2*a* for detail). Added topological location of *Tealliocaris* when included in the analysis (see electronic supplementary material, figure SF2*b* for detail). (*b*) Amended reconstruction of two *Oxyuropoda ligioides* in the Kiltorcan Old Quarry floodplains of the Upper Famennian (Upper Devonian), County Kilkenny, Leinster, Ireland. In association with the onland progymnosperms *Archeopteris hibernica*, and underwater algae *Bythotrephis* sp., the placoderms *Glyptolepis leptopterus*, and freshwater bivalve *Archanodon jukesi*. Reconstruction by Diane Dabir Moghaddam.

We investigated the phylogenetic position of †*Oxyuropoda* using a morphological dataset for malacostracan crustaceans modified from [7]. The phylogenetic significance of uropods in eumalacostracans was recently evidenced in [47]. Consequently, we scored four extra uropodal characters that are preserved in *Oxyuropoda*, following the approach of [48] (electronic supplementary material, dataset SD1). Likewise, because amphionidacean crustaceans were recently demonstrated to be decapod larvae instead of proper distinct taxa [12,48,49], the operational taxonomic unit (OTU) Amphionidacea was culled from our analyses. Final data matrices after [7] and [47] (25 OTUs with 181 adult morphological characters) were built in MorphoBank [50] (electronic supplementary material, dataset SD2; available at https://morphobank.org/permalink/?P3989). We included in a second analysis †*Tealliocaris*, a Late Devonian–Carboniferous eumalacostracan with debated peracarid or decapod affinities [12,51–56] that has been reported from late Fammenian (VCo Oppel biozone) freshwater (or at least continental water) horizons of Belgium [57,58]. Undetermined and not preserved characters were scored as ‘?’, and inapplicable characters as ‘—’. Multiple character states present in a given OTU were scored as polymorphisms*.* We analysed the dataset using Bayesian inference as implemented in MrBayes v. 3.2.6 [59]. The dataset was analysed under the traditional Mk model [60] with an ascertainment bias correction to account for scoring only variable morphological characters, and gamma distributed rate variation. Each analysis was performed with two independent runs of 3 × 10^7^ generations each. We used the default settings of four chains (one cold and three heated) per independent run. The relative burnin fraction was set to 25% and the chains were sampled every 200 generations. The temperature parameter was set to 0.01 as determined by preliminary runs to achieve chain mixing values in the optimal range (0.4–0.8). The convergence of independent runs was assessed through the average standard deviation of split frequencies ASDSF = 0.01 and potential scale reduction factors (PSRF ≈ 1 for all parameters [61]). We used Tracer v. 1.7.1 [62] to determine whether the runs reached the stationary phase and to ensure that the effective sample size (ESS) for each parameter was greater than 200. Results of the Bayesian runs were summarized as a majority-rule consensus tree of the post-burnin sample (figure 2; electronic supplementary material, figure SF2). The obtained tree branches were constrained over geological time in our figures, respectively, to estimated divergence age obtained or recommended from morphological and molecular studies on Branchiopoda [63], Isopoda [10,11,13] and Amphipoda [64].

## 3. Results and discussion

The advanced imaging of the only known *O. ligioides* specimen resolved both new anatomical features, never observed by optical microscopy, and the artefacts leading to previous erroneous interpretations of the fossil (figure 1*a–h*). Elongated elements have earlier been questioned to correspond either to antennae/uropod parts or to plant remains that look very similar to the animal appendages and are very abundant in the specimen [20,32]. Some of these are now surely assigned to plants observed detached from, and underneath, the arthropod body (black-and-white arrows in figure 1*b*). A three-dimensional surface rendering using digital microscopy accentuated a different extent of deformation between the right and left pleurae, with the left ones having bent more vertically than the right ones (electronic supplementary material, figure SF1*b*). Parallel to the left pleurae, the specimen exhibits a marked groove that we interpret as a break associated with the more pronounced compression of the left side of the thorax (figure 1*b*). This localized bending implies that the body of *Oxyuropoda* was generally flattened dorsoventrally, but had the extremities of its pleurae directed ventrally rather than laterally. Elements of the cephalon previously suggested to be the eyes are here revealed to be topographical artefacts (thick dash-lines in electronic supplementary material, figure SF1*e*), which we cannot exclude to reflect more ventrally located organs, but which we rather interpret as local, and asymmetric, bulges reflecting superposition of appendages. The first segments of the antennae—with large peduncles (figure 1*e,f*)—and the outlines of the buccal appendages are observed for the first time (figure 1*e,f*; electronic supplementary material; figure SF1*c–f*). These are comparable to the derived appendages of modern malacostraca, being elongated in shape (*pediform* following [65]) and geniculated, and we interpret them to be maxillipeds, maxillae (Mx1 and Mx2) and mandibles, as well as a probable pair of additional appendages that are hard to define in outline (figure 1*e,f*). The mandible structure shows a strong incisor, crenelated at its very tip end and bearing four teeth, as well as an elongated palp directed forward (figure 1*e,f*). The number of these appendages suggests that there are three somites forming the cephalon part of the cephalothorax (despite rather reduced cephalon dimensions, so likely thoracomeres 1 and 2 are fused, figure 1*a–d*); the presence/absence of a carapace above these segments cannot be established. The thorax comprises six thoracomeres (highlighted by short regions of overlapping proximal pleurae, figure 1*c*) increasing in size from anterior to posterior (figure 1*a–d*; electronic supplementary material, figure SF1*a*), each crossed by two transverse suture ridges and likely bearing a pair of pereopods the bases of which are locally visible (figure 1*c*; electronic supplementary material, figure SF1*c–f*). Some pleurae exhibit longitudinal to oblique grooves, evoking the coxal articulations found in Isopoda thoracomeres. Close examination of these structures reveals they correspond to be taphonomic cracks, meaning there is no anatomical evidence for coxal plate articulations in *Oxyuropoda* (electronic supplementary material, figure SF1*g*). The pleon is composed of six pleomeres (figure 1*a–d*; electronic supplementary material, figure SF1*a*), with the last one bearing the telson and a pair of uropods posteriorly oriented and styliform in shape (figure 1*g,h*). See electronic supplementary material, text ST1 for systematic palaeontology and full description, including measurements.

A cephalothorax, six pleonal segments (excluding telson) and uropods are malacostracan features [5,7,8,65–68], proper to all lineages but Leptostraca, while a stout tooth-like mandibular incisor is not found in these nor within Bathynellacea [8]. The absence of furcal extensions on the telson is also improper to Hoplocarida, these being indeed displayed in some extinct representatives [8]. The incorporation of a number of anterior thoracomeres into the cephalon is typical of all other malacostracan clades [5,7,8]. The body plan of *Oxyuropoda* displays only seven directly observable cephalothoracic somites out of the nine typical of malacostracans, requiring further digging into other diagnostic characters such as its uropodal features [47]. In eumalacostracan crustaceans, uropods represent a modification of the sixth pair of pleonal appendages, which, in contrast to caudal furca, are real, articulated appendages with a basipodite; they are posteriorly oriented and styliform in shape in *Oxyuropoda*. The basipodite is straight without extensions, and the uropod is without carinae (contrary to interpretation in [25], ridge corresponding to exo/endo-podites division), a combination of features found in Bathynellacea and in lately diverged peracarids (Spelaeogriphacea, Mictacea, Cumacea, Tanaidacea, Isopoda and Amphipoda) [8,47].

Bayesian inference phylogenetic analysis places *Oxyuropoda* among a strongly supported late diverged clade of Peracarida (posterior probability 83)—previously found in [6,7,69]—that includes Amphipoda, Cumacea, Tanaidacea and Isopoda (referred as clade ACTI; figure 2*a*; electronic supplementary material, figure SF2*a*). The internal phylogenetic relationships of these main peracarid clades, with the exception of a few more-often recovered patterns (e.g. Mysida grouped with Lophogastrida), are so far not resolved [4–9,13,65–67,69–74]. The input of *Oxyuropoda* maintains (i) the sister-relationship of Isopoda and Amphipoda recovered in [6–8,65,67,69,73,74], as well as (ii) the earliest divergence of the marine Mysidacea (Mysida and Lophogastrida) [5–8,65,67,69,71–74], followed by those of (iii) marine Thermosbaenacea and (iv) a mixed marine and continental clade comprising Spelaeogriphacea, ACTI and clade Mictacea/Bochusacea. *Tealliocaris*, whose affinities remain unresolved in our analysis, is found outside of modern peracarid clades (figure 2*a*; electronic supplementary material, figure SF2*b*). With its Late Devonian age, *Oxyuropoda* is the oldest known crown-group Peracarida, outdating *Anthracocaris scotica* Peach, 1882 from the Visean (Early Carboniferous) of the Calciferous Sandstone of the UK [14], which has been identified as a crown Tanaidacea (Anthracocarididae, see [15]). No stem-peracarid lineage has so far been identified [75]. Based on the information currently available, the molecular/fossil-based supported diversification times of amphipods and isopods on one side [10,11,64], and of branchiopods on the other [63], suggest a first peracarid diversification in the latest Cambrian (figure 2*a*). Recovering *Oxyuropoda* (two thoracomeres integrated in the cephalon and six in the thorax) as a crown peracarid may help polarize the direction of change of cephalic integration of anterior thoracomeres among Peracarida, yet this remains tentative until intrarelationships within crown Peracarida are better resolved.

Finally, the affinity of *Oxyruropoda* gives insight into the timing of colonization of non-marine environments by peracarids. *Oxyuropoda* plots either as the direct sister taxon, or within a sister clade (clade ACTI for Spelaeogriphacea) of groups with partial or strict freshwater representatives, the record of which even extends to their fossil representatives (e.g. Tanaidacea, Isopoda, Spelaeogriphacea) (figure 2*a*). Being likely part of an independent lineage of peracarids, *Oxyuropoda* provides further evidence that derived Peracarida were present in continental settings as early as the Fammenian, implying the fast colonization of continental waters (and land?) during the evolution of clade ACTI and likely of Spelaeogriphacea. Overall, our results indicate that besides Branchiopoda [57,76–80], and regardless of the affinities of tealliocaridids, highly derived vericrustacean groups had already colonized continental ecosystems by the Late Devonian (Famennian, more than 360 Ma) (figure 2*a*,*b*; electronic supplementary material, figure SF3).

## Data accessibility

Requests for access to the fossil material should be addressed to Nigel Monaghan (nmonaghan@museum.ie) of the National Museum of Ireland, Natural History, Dublin. Additional data are available as electronic supplementary material, as well as on MorphoBank to the link http://morphobank.org/permalink/?P3989.
